# Design of Self-Supported Flexible Nanostars MFe-LDH@ Carbon Xerogel-Modified Electrode for Methanol Oxidation

**DOI:** 10.3390/ma14185271

**Published:** 2021-09-13

**Authors:** Ghada M. Abdelrazek, Mohamed M. EL-Deeb, Ahmed A. Farghali, Agustín F. Pérez-Cadenas, Abdalla Abdelwahab

**Affiliations:** 1 Materials Science and Nanotechnology Department, Faculty of Postgraduate Studies for Advanced Sciences, Beni-Suef University, Beni-Suef 62511, Egypt; ghadaabdelrazek123@gmail.com (G.M.A.); farghali@psas.bsu.edu.eg (A.A.F.); aabdelwahab@psas.bsu.edu.eg (A.A.); 2 Chemistry Department, Faculty of Engineering, Basic Science, Misr University for Science and Technology (MUST), 6th of October City, Giza 12566, Egypt; 3 Applied Electrochemistry Laboratory, Chemistry Department, Faculty of Science, Beni-Suef University, Beni-Suef 62511, Egypt; 4 Carbon Materials Research Group, Department of Inorganic Chemistry, Faculty of Sciences, University of Granada, Campus Fuentenueva s/n, 18071 Granada, Spain; 5 Unit of Excellence in Chemistry Applied to Biomedicine and the Environment, University of Granada, 18071 Granada, Spain; 6 Faculty of Science, Galala University, Sokhna, Suez 43511, Egypt

**Keywords:** fuel cells, particle size, methanol oxidation, carbon xerogel

## Abstract

Layered double hydroxides (LDHs) have emerged as promising electrodes materials for the methanol oxidation reaction. Here, we report on the preparation of different LDHs with the hydrothermal process. The effect of the divalent cation (i.e., Ni, Co, and Zn) on the electrochemical performance of methanol oxidation was investigated. Moreover, nanocomposites of LDHs and carbon xerogels (CX) supported on nickel foam (NF) substrate were prepared to investigate the role of carbon xerogel. The results show that NiFe-LDH/CX/NF is an efficient electrocatalyst for methanol oxidation with a current density that reaches 400 mA·m^−2^ compared to 250 and 90 mA·cm^−2^ for NiFe-LDH/NF and NF, respectively. In addition, all LDH/CX/NF nanocomposites show excellent stability for methanol oxidation. A clear relationship is observed between the electrodes crystallite size and their activity to methanol oxidation. The smaller the crystallite size, the higher the current density delivered. Additionally, the presence of carbon xerogel in the nanocomposites offer 3D interconnected micro/mesopores, which facilitate both mass and electron transport.

## 1. Introduction

The fast consumption of fossil fuels and constantly growing environmental issues have promoted major research interests in developing clean, reliable, and sustainable technologies for energy conversion, such as fuel cells [1,2]. From this point of view, direct methanol fuel cells (DMFC) have attracted considerable attention for mobile and stationary electronic devices because of the high energy density of methanol, low operating temperature, high performance, low cost, easy storage, and low pollutant emissions [3,4]. Because of the possible uses of lower-cost non-platinum catalysts to achieve higher methanol oxidation, alkaline DMFCs are considered more interesting than acidic ones due to the less corrosive alkaline environment that improves their durability [5]. Methanol is preferable to ethanol as a fuel due to its high energy density, easy storage, preparation, and availability [6]. Currently, the two most significant technological barriers to DMFCs development are the low catalytic activity of anode (sluggish anode reaction) [7] and “methanol cross-over” to the cathode [8]. The latter problem emerges from the fact that methanol diffuses through the electrolytes and interacts directly with oxygen at the cathode, greatly reducing the efficiency of the used methanol and losing power of the fuel cell [9]. A variety of noble metals have been used as catalysts for electrocatalytic methanol oxidation, such as Pt and Pt-based alloys [10,11]. However, they suffer from high cost, low reserves, and extreme poisoning by the intermediates, such as CO_2_ [12]. Thus, current research has been aimed at developing new low-cost and highly-active catalyst materials for methanol oxidation reaction (MOR) [13]. The literature offers an overview of catalysts for DMFCs such as metal oxide [14], carbon nanotubes as a support material [15,16], metal organic framework and their composites [17]. Nanomaterials are interesting in the electrochemical field owing to their high surface area, unique structure, and highly promising physicochemical properties. Among a range of hydroxides and oxides, layered double hydroxides (LDHs) are a class of two-dimensional (2D) anionic clays made up of positively charged brucite-like host layers and interlayer anions of exchangeable charge balancing to obtain electro-neutrality [18,19,20,21]. Their general formula: [M^II^_1−x_ M^III^_x_(OH)_2_]^x+^[A^n−^]_x/n_·yH_2_O, where M^II^ and M^III^ are arranged in the LDHs layers as divalent and trivalent metal cations, A^n−^ is an n-valent anion, and y is the number of water molecules in the interlayer [22]. Recently, due to their possible technical applications in areas such as separation, catalysis, sensors, and electrochemistry, these layered materials have recently received considerable attention due to desirable properties, including well anion exchange, high surface area, and low cost [23,24]. These attractive features enhance more exposed active sites and mass or ion transport, which are useful for improving the electrocatalytic activity, which clearly drives them as alternative materials to catalyze the oxidation of methanol. In addition, the development of surface porosity in nanosheets enhances the diffusion of ionic reagents, and the rise of unsaturated coordination sites can be accomplished [25].

Unfortunately, LDH aggregation and low electrical conductivity decreases its stability and suppresses its electrochemical activity [26]. Consequently, a combination of LDH with conductive support can reduce the agglomeration of LDH and promote its electrocatalytic activity [27]. Several studies found a relationship between the material particle size and its activity in a certain electrochemical application [28,29,30]. In this context, it is important to decrease the composite particle size for better electrochemical performance [31]. For example, Chae, Gyu S. et al. [30] prepared nanostructured FeS dispersed onto N, S dual-doped carbon nanotube–graphene composite support as electrocatalyst for the oxygen reduction reaction. In this study, a synergy effect between the composite particle size and its activity was observed, i.e., the lower the particle size, the higher the produced activity.

Therefore, combining LDH with conductive substrate materials, such as carbon materials, can effectively overcome these problems. The support materials include graphene, carbon nanotube, carbon quantum dots, and carbon xerogel (CX). Carbon xerogel is a novel nanostructured carbon material that has excellent properties of high specific surface area, high porosity, controllable pore size, high density, and high conductivity [32,33]. It can be obtained in different forms, and its structure can be controlled according to the synthesis and processing conditions. Therefore, CX was used as a support material in various applications such as catalysis [34], electro-catalysis [35], adsorption [36], and energy storage [37]. Wang et al. [21] reported that NiAl-LDH/AuNPs/GCE displays a higher catalytic activity for methanol oxidation. Compared to NiAl-LDH without AuNP, the reinforcement can be related to the synergistic effect between them. Furthermore, according to [38], hierarchical flower catalysts Au/NiAl-LDH were prepared for the selectivity of alcohols oxidation, and it was observed that the hierarchical pores showed activity greater than the common Au/NiAl-LDH, which attributed to the shape selectivity of macropores.

Li et al. [39] demonstrated that hierarchical nanoarrays of MFe-LDH, i.e., M = Ni, Co, and Li, show highly electrocatalytic performance towards different oxidation reactions of small molecules, i.e., water, hydrazine, methanol, and ethanol. The resulting arrays of NiFe-LDH show promising behavior in the oxygen evolution reaction (OER).

Vlamidis et al. [18] demonstrated that NiFe-LDHs can achieve high activity for the oxidation of methanol as a consequence of the role played by Fe in the electrocatalytic process.

Additionally, Jia et al. [13] synthesized NiFe/LDH and NiFe/LDH@MnO_2_ nanosheets for ethanol oxidation. The LDH@MnO_2_ showed excellent catalytic activity for oxidation of ethanol compared to NiFe. The enhancement could be attributed to MnO_2_, which increases the concentration of OH ads-species on the surface of Ni-Fe LDH. Gamil et al. [40] prepared NiCr-LDH@rGO nanocomposite with different concentrations of reduced graphene oxide, which showed much higher activity for the electro-oxidation of methanol than pure NiCr-LDH. Eldeeb et al. [35] fabricated nickel cobaltite/CX nanocomposite by hydrothermal method. The data showed that the NiCo_2_O_4_/CX provides higher catalytic activity compared to pure NiCo_2_O_4_, which is attributed to the synergistic effect of the pore geometry. Nickel Foam (NF) is an attractive current collector commonly used in energy storage and energy conversion devices due to the good electrocatalytic activity of Ni and the properties such as the high surface area, high porosity that promotes the mass transfer of the reactants, excellent mechanical strength, electrical conductivity, and resistance of corrosion [41].

Direct deposition of active material on the NF facilitates contact between the electrode material and the current collector, which improves ion transport and electrochemical performance [42]. Yu et al. [43] reported greater electro-oxidation of methanol and stability of mesoporous NiCo_2_O_4_ nanorods with graphene on nickel foam by a chemical vapor deposition method. Roy et al. [44] prepared cobalt hydroxide Co(OH)_2_ nanoflakes on Ni foam by an electroplating technique. The results showed that Co(OH)_2_ has excellent electrocatalytic activity towards methanol oxidation, and this activity is attributed to the direct growth of an electroactive nanostructure which improves mechanical adhesion and facilitates the transfer of electrons between the current collector and the electrocatalyst.

The current challenge with methanol electro-oxidation is to develop promising inexpensive electrode materials with excellent efficiency. The nanocomposites of Layered double hydroxides and carbon xerogels have not been studied before as electrocatalysts for methanol electro-oxidation. Therefore, in this work, six nanohybrids composites of Layered double hydroxides supported on nickel foam were prepared by the hydrothermal process with and without carbon xerogels to investigate the role of carbon xerogel on the electrode activity. Moreover, the effect of the divalent cation on the electrochemical activity of methanol oxidation was studied by using three divalent cations, i.e., M^II^ = Ni, Co, and Zn, while the used trivalent cation Fe was fixed. The prepared nanocomposites were denoted as NiFe-LDH/NF, NiFe-LDH/CX/NF, CoFe-LDH/NF, CoFe-LDH/CX/NF, ZnFe-LDH/NF, and ZnFe-LDH/CX/NF.

## 2. Materials and Methods

### 2.1. Materials

Nickel nitrate Ni(NO_3_)_2_·6H_2_O, iron nitrate Fe(NO_3_)_3_·9H_2_O, cobalt nitrate Co(NO_3_)_2_·6H_2_O, zinc nitrate Zn(NO_3_)_2_·6H_2_O, nickel acetate (Ni(Ac)_2_·6H_2_O, urea, resorcinol, and formaldehyde were all used without further purification. Deionized (DI) water was used for synthesis and treatment processes under ambient conditions. Ni foam was used as a substrate and current collector. It was sonicated using 1M HCl solution followed by acetone, ethanol, and distilled water for 10 min each in sequence to remove the oxide layer. The supporting electrolyte for all electrochemical experiments was 1 M KOH.

### 2.2. Preparation of Carbon Xerogels (CX)

CX is prepared by sol-gel as previously reported [34]. In short, resorcinol (R) and formaldehyde (F) were dissolved in water (W) in the presence of nickel acetate instead of alkaline catalyst to improve the resulting surface area. The weight of the metal catalyst was calculated to be 6 wt.% of the produced CX. The molar ratios used for R/F and R/W were 0.5 and 0.06, respectively. After preparing the initial mixture, the mixture was subjected to stirring, then poured into glass molds. The curing and gelation temperature was 40 °C for one day, then 80 °C for five days. The organic gels produced were subjected to microwave drying to obtain their corresponding organic xerogels. Finally, CX was obtained by carbonizing organic xerogels at 900 °C for 2 h with a heating rate of 5 °C/min in the presence of N_2_ gas.

### 2.3. Preparation of Ni-Fe LDH Nanosheet Array on NF Substrate

Nickel-iron layered double hydroxide (NiFe-LDH) nanosheet array on Ni foam was synthesized via a simple hydrothermal process [45]. Typically, Ni(NO_3_)_2_·6H_2_O (0.345 g), Fe(NO_3_)_3_·6H_2_O (0.159 g), and urea (0.166 g) were dissolved in 160 mL of deionized water. The mixture was stirred at room temperature for 15 min to form a clear solution and then transferred to a 200 mL stainless steel Teflon autoclave. A pretreated piece of NF was dipped into the reaction solution. The autoclave was tightly closed and kept at 100 °C for 24 h in an electronic oven. After naturally cooling to room temperature, the coated NF was washed several times with distilled water and absolute ethanol. For comparison, CoFe-LDH and ZnFe-LDH/Ni foam nanosheet arrays were synthesized with the same procedure.

### 2.4. Preparation of Ni-Fe LDH Nanosheet Array/CX on NF Substrate

An amount of 40 mg of CX was dispersed in 30 mL of ethanol using an ultrasonic probe (SONICS, Probe diameter 5–8 mm, pulse, on/off 9.9 and 3 s, Newtown, CT, USA) for 15 min. Then, a solution containing Ni(NO_3_)_2_·6H_2_O (0.345 g), Fe(NO_3_)_3_·6H_2_O (0.159 g), and urea (0.166 g) in 130 mL of H_2_O was added and ultrasonicated for 15 min. A piece of the pretreated Ni foam (1 cm × 5.5 cm) was then immersed in a 200 mL Teflon-lined autoclave containing the above mixture solution. The autoclave was sealed, kept at 100 °C for 24 h, and then allowed to cool naturally to room temperature after being washed several times with distilled water and ethanol and dried at 60 °C for 6 h. Co-Fe LDH/CX/NF and Zn-Fe LDH/CX/NF were synthesized with the same procedure.

### 2.5. Characterization

The structures of the prepared materials were examined using a transmission electron microscope HRTEM (JEOL JEM 2100, JEOL, Tokyo, Japan), and morphologies were checked using the scanning electron microscope SEM (FEI-Quanta FEG-250 SEM, Basel, Switzerland). The phase identification and crystallinity were analyzed using X-ray diffraction (PANalytical Empyrean, Eindhoven, Netherlands) with CuKa radiation (wavelength 1.54045 Å), accelerating voltage of 40 KV and a current of 35 mA. Fourier transformation infrared spectroscopy (FTIR) was performed to obtain information about the intercalation of LDH using Vertex 70 (Bruker, Berlin, Germany). Raman spectroscopy (FT-Raman) of the as-prepared composites was obtained with Bruker Senterra Raman Microscope (Bruker Optics Inc., Germany). The BET surface area and pore size distribution were determined using a surface area analyzer (TriStar II 3020, Micromeritics, Norcross, GA, USA).

### 2.6. Electrochemical Measurements

Electrochemical measurements were carried out using NOVA 1.11 software Potentiostat/Galvanostat (AUTOLAB PGSTAT 302N, Metrohm, Herisau, Switzerland) in a three-compartment glass cell with electroplated film on nickel foam as working electrode, Pt as counter electrode, and Ag/AgCl as reference electrode at room temperature. An aqueous solution of 1 M of KOH with and without 0.5 M of methanol was used as the electrolyte to study the electrocatalytic activity of the prepared electrodes. The cyclic voltammetry (CV) measurements for each electrode were performed at different scan rates within the potential range of 0–0.7 V. Chronoamperometry (CA) tests were performed at 0.7 V for 3600 s. Furthermore, the electrochemical impedance spectroscopy (EIS) spectra were taken in the frequency range from 100 kHz to 0.01 Hz.

## 3. Results and Discussions

### 3.1. Physicochemical Properties

**Figure 1:**
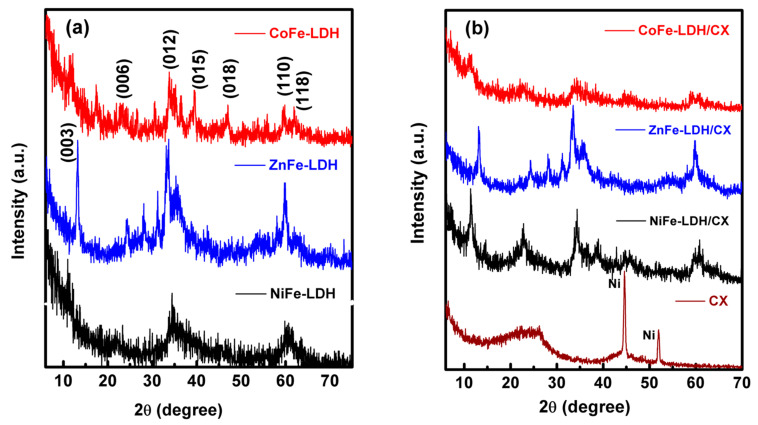
The XRD patterns of (**a**) XFe-LDH/NF and (**b**) XFe-LDH/CX/NF.

Powder X-ray diffraction (XRD) was conducted to confirm the structure of the synthesized materials. The XRD pattern of the prepared LDHs with different divalent cations is presented in Figure 1a. The reflection peaks of the layered double hydroxide structure are clearly seen. The diffraction peaks at 2θ of 11.6°, 23.2°, 34.5°, 39.1°, 46.5°, 59.9°, and 60.9° could be assigned to the (003), (006), (012), (009), (015), (018), (110), and (113) plane reflections, respectively. The prepared LDHs possess a Rhombohedral crystal system with a space group of R-3 (ICDD card no. 01-082-8040) [46,47]. Figure 1b shows the XRD pattern of the carbon xerogel, which has a diffraction peak at 2θ of 26° representing the (002) diffraction plane of graphite, and the two main peaks at 2θ of 44° and 51° are assigned for the metallic nickel that has been used as a catalyst during its preparation. It should be noted that the diffraction peak of amorphous CX at 2θ of 26° overlaps with the (006) diffraction plane peak of NiFe-LDH, so it is difficult to be distinguished in the composites. The increase in the graphitic order induces an increase in the nanocomposites crystallinity, which enhances their conductivities; this behavior is clearly seen for NiFe-LDH/CX with the presence of a diffraction peak centered at 2θ of 26° [48]. Table 1 represents the average crystallite sizes for the prepared nanocomposites calculated using Scherrer’s equation. The increase in the crystallite size is considered evidence for the intercalation of carbon xerogel between the LDH layers. It is apparent that the crystallite size of NiFe-LDF is the least when compared with CoFe-LDF or ZnFe-LDF. Additionally, its composite with carbon xerogel NiFe-LDH/CX has the least crystallite size than CoFe-LDH/CX or ZnFe-LDH/CX. Many publications found a relation between the crystallite size and the activity of the composites for certain applications [49,50].

**Figure 2:**
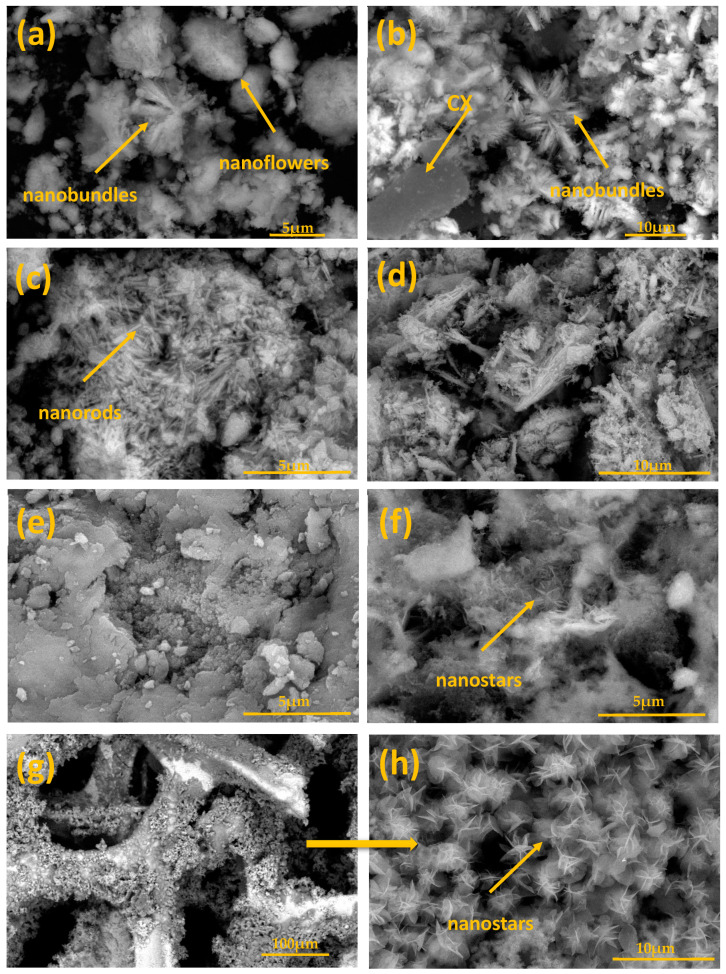
SEM images of (**a**) ZnFe-LDH, (**b**) ZnFe-LDH/CX, (**c**) CoFe-LDH, (**d**) CoFe-LDH/CX, (**e**) NiFe-LDH, (**f**) NiFe-LDH/CX, and (**g**,**h**) NiFe-LDH/CX/NF.

**Figure 3:**
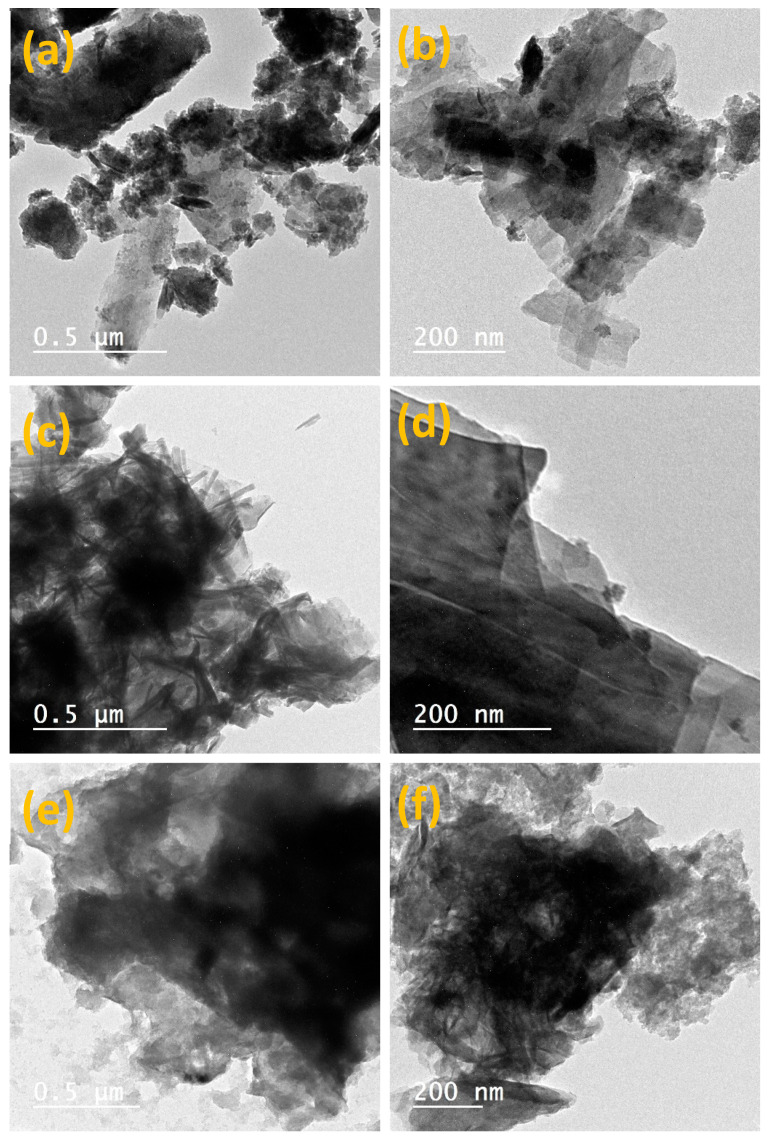
HR-TEM images of (**a**) ZnFe-LDH, (**b**) ZnFe-LDH/CX, (**c**) CoFe-LDH, (**d**) CoFe-LDH/CX, (**e**) NiFe-LDH, and (**f**) NiFe-LDH/CX.

The scanning electron microscopy (SEM) images of the nanocomposites are presented in Figure 2. It is apparent that different morphologies were observed for the prepared LDHs with different divalent cations. In Figure 2a, in addition to the presence of ZnFe-LDH in the layered structure, other two morphologies were observed, namely, nanobundles and nanoflowers with a three-dimensional hierarchical structure, which is also confirmed with TEM, Figure 3a. This morphology was maintained with the incorporation of carbon xerogel in ZnFe-LDH, i.e., ZnFe-LDH/CX, Figure 2b and Figure 3b, in which the CX particles are anchored on the ZnFe-LDH flowers and filled the interstitial space of the flowers.

The SEM image for CoFe-LDH, Figure 2c, shows that the LDH has a nanorod structure that has stacked to each other to form sheets and bundles, as also confirmed by TEM Figure 3c, which is in agreement with [51]. With the incorporation of CX into the CoFe-LDH, Figure 2d and Figure 3d, the CX is intercalated between the LDH layers, and the CX has been successfully coated on the surface of CoFe-LDH where the nanorod structures can still be observed. It is also clearly shown in Figure 2e that the NiFe-LDH has a continuous structure of interconnected particles, and a proprietary plate-like morphology can be obtained. TEM micrograph for NiFe-LDH, Figure 3e, shows that the morphology of the prepared NiFe-LDH is similar to a plate and uniform structure in nature [47]. Moreover, the combination between NiFe-LDH and CX improved the structure and formed a new morphology, Figure 2 and Figure 3f, SEM and TEM images which show a nanostar structure for NiFe-LDH/CX. Figure 2g–h shows the SEM image for the deposition of NiFe-LDH/CX onto the surface of nickel foam; NiFe-LDH/CX/NF, the intrinsic 3D structure of the Ni foam was well maintained and uniformly covered by NiFe-LDH/CX nanoparticles. For most of these particles, many branches have been developed to give them a star shape. This type of branched particles presumably results from the overgrowth of the initially formed faceted particles. Li et al. suggest a relationship between the materials morphology and their electrochemical activity for methanol electro-oxidation [52].

**Figure 4:**
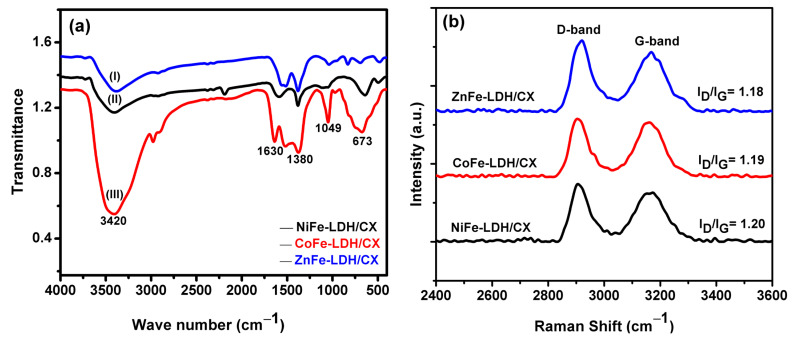
(**a**) FTIR spectra of hybrid (I) ZnFe-LDH/CX, (II) NiFe-LDH/CX, and (III) CoFe-LDH/CX, and (**b**) FT-Raman.

The chemical composition and main functional groups of nanohybrids were studied by FTIR in the region of 400–4000 cm^−1^, as observed in Figure 4a. The wide band that appeared at 3420 cm^−1^ is related to the OH-stretching band of the metal hydroxyl groups and the hydrogen-bonded interlayer of the water molecules [22]. Additionally, the band at 1630 is attributed to the bending mode of the OH group of water molecules [40]. The peak located at 1380 cm^−1^ is assigned to the stretching vibrations of N–O of NO_3_^−^ groups, confirming the intercalation of NO_3_^-^ anions [53]. The peak at 1049 cm^−1^ corresponds to the stretching vibration of C–O [54]. The characteristic bands at 673 is related to metal–OH bending (Hou et al. 2018a; Jin et al. 2016) [55], confirming the existence of the LDH in the composites.

In Raman spectra for the prepared LDHs/CX, Figure 4b, there are two strong peaks at about 1338 and 1595 cm^−1^, which could be assigned to the D and G bands of carbon, respectively [56]. These data confirm the coexistence of LDHs and carbon xerogel in the composites. The D band is associated with the alternating ring vibrations in condensed benzene rings, while the G band is associated with the development of the sp^2^ carbon structure during the carbanization process [34]. The ratio of the intensity of I_D_/I_G_ was calculated for the LDHs/CX, Figure 4b. As it can be seen, the ratio between I_D_/I_G_ for NiFe-LDH/CX is the highest one compared to the other two samples, indicating a more disordered structure due to the reduction of the sp^2^ region size.

**Figure 5:**
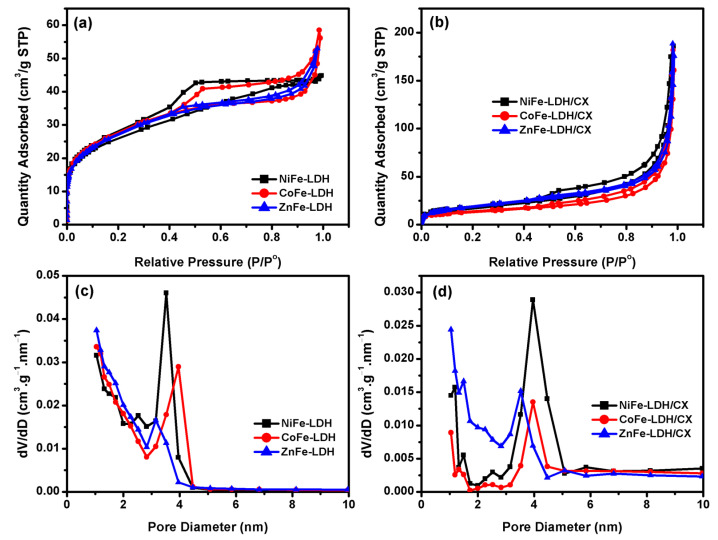
N_2_ adsorption–desorption isotherms for (**a**) XFe-LDH, (**b**) XFe-LDH/CX, (**c**) and (**d**) BJH pore size distribution plots of the as-prepared materials.

The N_2_ adsorption–desorption isotherms and pore size distributions are represented in Figure 5. Based on IUPAC classification [57], the isotherms for all electrocatalysts are combinations of type I and type IV isotherms [58], indicating that the prepared composite’s pores have mainly microporous character and less contribution of mesoporous one. In Figure 5a, there is a higher uptake of N_2_ at lower relative pressure, indicating micropores filling. Moreover, NiFe-LDH and CoFe-LDH have hysteresis loops of type H4 at a moderate relative pressure; narrow slit-like pores in the samples or the internal particles voids have an irregular shape with broad size distributions, as suggested by the BJH method applied to the obtained isotherms, Figure 5c [59].

The N_2_ isotherms for LDHs/CX, Figure 5b, are also a combination of type I and IV with an increase in the gas adsorption at high relative pressures, which suggests the development of meso–macropore diameter upon intercalation of carbon xerogel between the LDH layers, Figure 5d. The texture characteristics for the samples are shown in Table 2. As it can be seen, there is a decrease in the BET specific surface area (S_BET_) up on introducing the carbon xerogel between the LDHs layers, which is a confirmation with the development of mesoporous character as observed from the increase in their pore diameter. These hierarchical pore structures are considered to be beneficial in electrocatalytic applications due to minimizing the diffusion barriers [60]. Figure 5c,d shows the BJH pore size distribution for the as-prepared sample. In Figure 5c, the pore size distributions for LDHs without carbon xerogel are a combination of micro–meso pores with an excess contribution of micropores except for NiFe-LDH. This ratio between micro–meso pores was changed with the incorporation of carbon xerogel into the LDHs structure with the increase in the mesopore diameter, Figure 5d. The textural characteristics of the samples are collected in Table 2.

### 3.2. Electrochemical Activity for Methanol Oxidation

**Figure 6:**
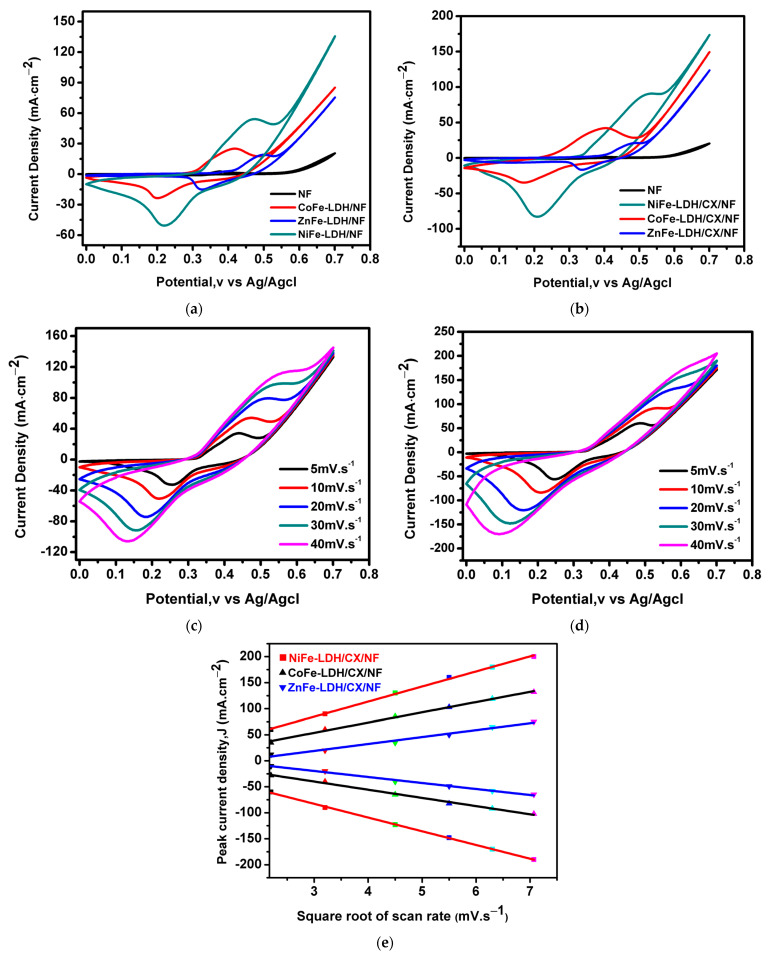
CVs at 1M KOH of (**a**) LDHs/NF at 10 mV/s, (**b**) LDHs/CX/NF at 10 mV/s, (**c**) NiFe-LDH/NF at different scan rates, and (**d**) NiFe-LDH/CX/NF at different scan rates. (**e**) Linear relationship between the forward/backward anodic peak currents and the square root of the scan rate for all nanohybrids with CX.

The electrocatalytic performance of the electrocatalysts was investigated using CV, CA, and EIS measurements. Cyclic voltammetry of the prepared nanohybrid electrodes was performed in 1M KOH within the potential range of 0–0.7 V (vs. Ag/AgCl) at different scan rates (5, 10, 20, 30, and 40 mV/s). Figure 6a,b shows cyclic voltammograms of the prepared nanocomposites at a scan rate of 10 mV·s^−1^. Figure 6a shows the cyclic voltammograms of LDHs supported on nickel foam (NF) in which all nanocomposites exhibit higher electrochemical activity than nickel foam alone. Additionally, the highest current density is obtained for NiFe-LDH/NF of 135.8 mA·cm^−2^. In addition, introducing carbon xerogel into the LDHs structure improve the electrochemical activity for the composites with the same order, and the current density for NiFe-LDH/CX/NF reaches 172.6 mA·cm^−2^, Figure 6b. This observation explains the role of carbon xerogel in developing the activity of the prepared catalysts by introducing new channels for ionic diffusion. It is also apparent that pairs of anodic and cathodic redox peaks are obtained for all CV curves, which are mainly attributed to charge transfer processes of solid-state redox [61].

The CV for NiFe-LDH/NF and NiFe-LDH/CX/NF at different scan rates are presented in Figure 6c,d, respectively. The increase in current density was noticed on various potential sweeps, implying the formation of an active layer over the catalyst created by the CV cycle. This is likely to be related to the entry of hydroxyl anions into the intermediate layer of the catalyst [62,63]. The linear relationship of the square root of the scan rate ranging from 5 to 40 mV/s with the anodic and cathodic current densities of the redox peaks is shown in Figure 6e. There is an increase in redox reactions with changing the scan rate, and the electrochemical process is controlled by the spread of OH in the mesopore cavity [35].

**Figure 7:**
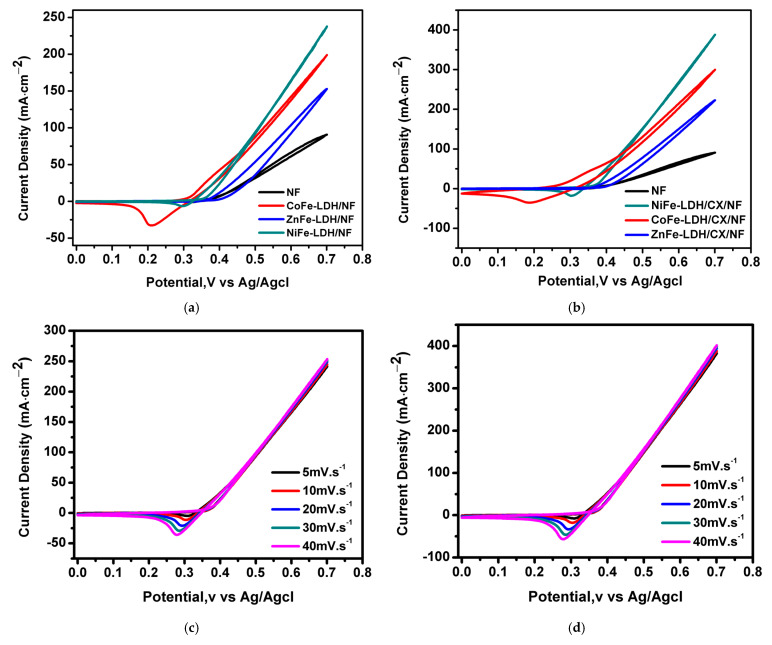

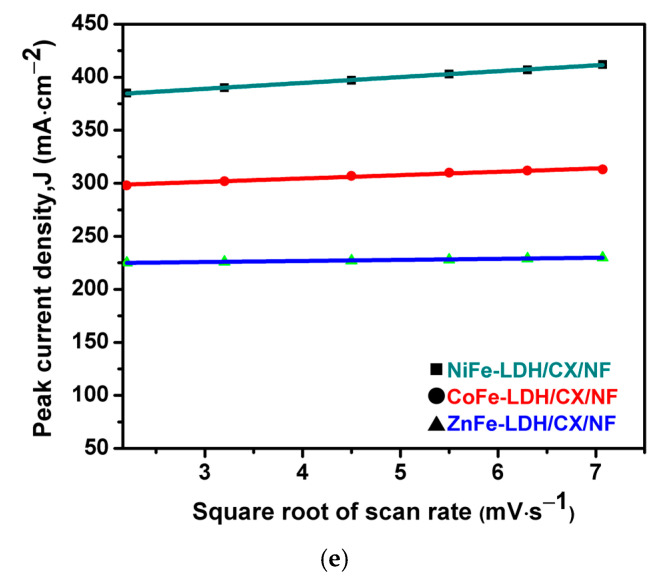
CVs at 1 M KOH + 0.5 M MeOH of (**a**) LDHs/NF at 10 mV/s, (**b**) LDHs/CX/NF at 10 mV/s, (**c**) NiFe-LDH/NF at different scan rates, and (**d**) NiFe-LDH/CX/NF at different scan rates. (**e**) Linear relationship between the anodic peak currents and the square root of the scan rate for all nanohybrids with CX.

Figure 7a shows the CV curves of the electrodes in 1 M KOH and 0.5 M MeOH at a scan rate of 10 mV/s. The electrochemical characteristics indicate that there is an increase in the current density after the addition of methanol, indicating oxidation of methanol on the surface of the prepared hybrids. Moreover, LDHs/CX composites have a higher activity to methanol oxidation due to the higher surface area delivered by the addition of CX to the composites, Figure 7b. The CV curves for NiFe-LDH/NF and NiFe-LDH/CX/NF at different scan rates are shown in Figure 7c,d, respectively. A higher anodic oxidation current density for the electro-oxidation of methanol reaches 400 mA·cm^–2^ at the NiFe-LDH/CX/NF compared to 90 mA·cm^–2^ at NF, Figure 7b, indicating a greater electrocatalytic response of NiFe-LDH/CX/NF electrode for electro-oxidation of methanol, which assures that CX can reduce agglomeration of LDH and form new material with higher electrochemical activity [35].

Interestingly, there was a relationship between the electrocatalytic performance of the nanohybrid LDH for the oxidation of methanol and the type of divalent cation. The better performance of the NiFe-LDH/CX/NF electrode has been related to the intrinsic activity of this compound, combined with its mesoporous structure and wide pore width that improve mass transport and transfer of electrons. Moreover, according to XRD data, NiFe-LDH/CX has the least average crystallite size among the nanocomposites of LDHs/CX. Different studies found a relationship between electrode activity and its crystallite size [49]. The literature has reported that defective Ni(OH)_2_ species promote OH^–^ switching from Ni(OH)_2_ to adjacent nanocrystals by forming new channels and active sites for OH^–^ adsorption and developing removal of carbon species, CO, which poisons the surfaces of the catalyst and increases the electrocatalytic activity of the LDH nanohybrid [64]. Figure 7e exhibits a linear relationship between the square root of the scan rate 5–40 mV·s^−1^ and the catalytic current density. The results indicate that the electro-oxidation of methanol on the NiFe-LDH/CX/NF is a surface controlled process [65]. The highly electroactive response of NiFe-LDH/CX/NF when comparing it with NiFe-LDH/NF is inferred from its high anodic and cathodic current densities. This enhancement in the electrocatalytic activity is observed although the specific surface area and cumulative pore volume of other nanohybrids are higher than those of NiFe-LDH/CX/NF, Table 2, and the enhancement in the electrocatalytic activity of our modified NiFe-LDH/CX/NF can also be attributed to the synergistic effect between NiFe-LDH and CX during the preparation of the nanocomposites, low crystallite size, and its unique nanostars morphology.

Interestingly, the electrocatalytic activity of our modified NiFe-LDH/CX/NF mesoporous electrode is higher than that previously reported for the electro-oxidation of methanol [66], as presented in Table 3. Most of the reported studies for methanol oxidation explain the activity of the electrocatalysts to the surface area of the catalyst. The catalyst with a higher surface area has higher activity for the oxidation of methanol. Although our new NiFe-LDH/CX/NF catalyst has a small specific surface area compared to the other reported electrodes, it has an excellent activity for MOR. The correlation between pore geometry, crystallite size, and morphology also has a great influence on electrode activity.

**Figure 8:**
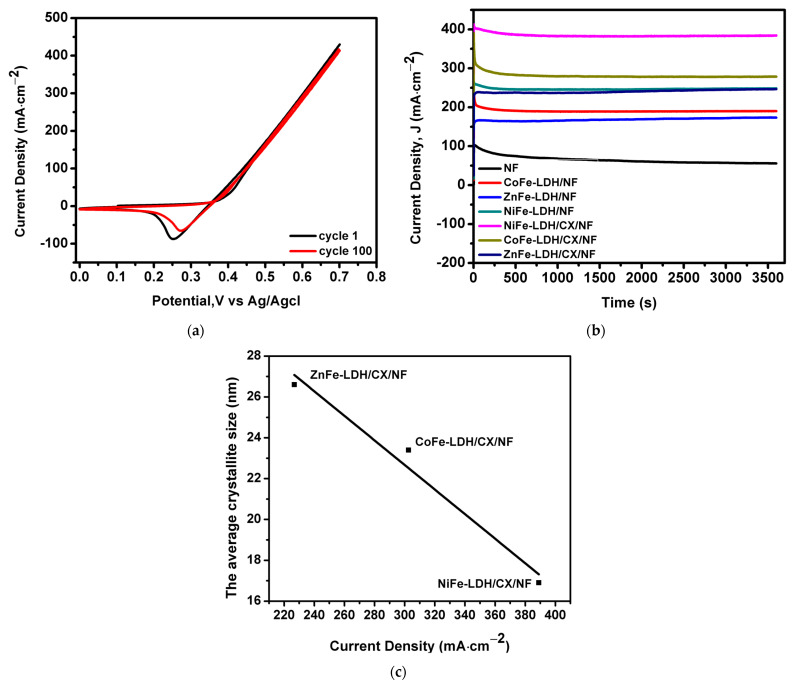
(**a**) Repetitive 100th cycle of NiFe-LDH/CX/NF in 1.0 M KOH + 0.5 M CH_3_OH at a scan rate of 40 mV/s. (**b**) Chronoamperometric response of the synthesized electrocatalysts in 1.0 M KOH + 0.5 M CH_3_OH at 0.7 V and (**c**) the crystallite particle size of the modified electrodes versus their electrochemical activity.

The electrochemical stability of the prepared materials is another important issue to consider in practical applications. Figure 8a shows the repetitive 100-cycle test for NiFe-LDH/CX/NF at a scan rate of 50 mV·s^−1^ in 1.0M KOH with 0.5 M CH_3_OH solution at room temperature. There is almost no change in the electrocatalytic activity towards the oxidation of methanol that can be observed after 100 cycles, which indicates exceptional stability for this nanohybrid.

Figure 8b characterizes the chrono-amperometric curves of nanohybrids in 1M KOH and 0.5 M methanol at a constant potential of 0.6 V vs. Ag/AgCl for 1 h. It can be seen that the obtained current densities for all catalytic systems are nearly stable after 3600 s. This could be assigned to the formation of reactive intermediates such as CO and COH during the oxidation reaction of methanol [67]. During the test process, all electrocatalysts were stable, but it was noted that the current density obtained with the NiFe-LDH/CX/NF catalyst was higher than others. This long-term stability could be attributed to its mesoporous structure, which causes the fast transformation of the adsorbed intermediates into products that increase the catalytic activity of the nanohybrids [10,68].

On the other hand, taking into account the effect of the crystallite size of the prepared materials on the electrochemical activity to methanol oxidation (Table 1), a clear relationship can be observed: the smaller the crystallite size, the higher the current density delivered, Figure 8c. In our opinion, the results of this work exhibit clear evidence that crystallite size, electrode morphology, and mesopore diameter development are crucial parameters involved in the electro-oxidation of methanol.

## 4. Conclusions

Series of layered double hydroxides with different divalent cations of Ni, Co, and Zn- iron LDHs were successfully prepared. All electrocatalysts show a very good activity to methanol oxidation, where NiFe-LDH/NF is the best one. Furthermore, introducing carbon xerogel into the LDHs structure significantly improves the electrocatalysts activity to methanol electrooxidation. This development can be attributed to the 3D structure of carbon xerogel, which facilitates the ionic diffusion inside the carbon matrix, resulting in higher OH^–^ adsorption. NiFe-LDH/CX/NF is the best catalyst for methanol oxidation with a current density of 400 mA·cm^–2^ with excellent stability. Additionally, there is a strong influence of the electrocatalysts crystallite size on the activity of methanol oxidation. The smaller the crystallite size, the higher current densities were obtained. Moreover, the morphology change of the electrocatalysts with the divalent cations has an influence on their activity towards MOR. These results suggest the potential use of LDHs/CX/NF electrocatalysts in proton-exchange membrane fuel cells because they are inexpensive and have high activity and excellent stability.

## Figures and Tables

**Table 1:** Crystallite size of the synthesized LDHs.

Sample Name	The Average Crystallite Size (nm)
NiFe-LDH	15.9
CoFe-LDH	17.3
ZnFe-LDH	18.7
NiFe-LDH/CX	16.9
CoFe-LDH/CX	23.4
ZnFe-LDH/CX	26.6

**Table 2:** Textural data of the prepared materials.

Sample	S_BET_	Pore Volume	Mean Pore Diameter
	m^2^/g	cm^3^/g	nm
NiFe-LDH	98.6	0.07	2.24
CoFe-LDH	101.2	0.09	3.60
ZnFe-LDH	99.1	0.11	4.78
NiFe-LDH/CX	47.1	0.28	23.37
CoFe-LDH/CX	64.4	0.34	18.16
ZnFe-LDH/CX	68.6	0.32	15.93

**Table 3:** Comparison of current densities, electrolyte, and applied potential with several reported electrocatalysts for methanol oxidation.

Electrocatalyst	Electrolyte	Applied Voltage	Current Density	Ref.
NiFe-LDH/CX/NF	1.0 M KOH/0.5 M CH_3_OH	0–0.7 V vs. Ag/AgCl	400 mA·cm^−2^	This work
3D NiCr-LDH/rGO	1.0 M KOH/3.0 M CH_3_OH	0–0.6 V vs. Ag/AgCl	2.2 mA·cm^−2^	[27]
Microsphere Co_3_O_4_/NF	1.0 M KOH/0.5 M CH_3_OH	0–0.6 V vs. Hg/HgO	36 A·g^−1^	[59]
Co(OH)_2_/NF	1.0 M KOH/0.5 M CH_3_OH	0–0.5 V vs. SCE	150 A·g^−1^	[44]
Mn doped Ni(OH)_2_	1.0 M KOH/0.5 M CH_3_OH	1–2 V vs. RHE	20 A·g^−1^	[3]
3D nickel networks	1.0 M NaOH/1.0 M CH_3_OH	0–0.8 V vs. Hg/HgO	175 mA·cm^−2^	[12]
NiCo_2_O_4_/CX	1.0 M KOH/0.5 M CH_3_OH	0–0.6 V vs. Ag/AgCl	98 mA·cm^−2^	[35]

## Data Availability

The data presented in this study are available on request from the corresponding author.
